# Association of perceived stress and coping strategies with the renal function in middle-aged and older Japanese men and women

**DOI:** 10.1038/s41598-021-04324-2

**Published:** 2022-01-07

**Authors:** Kayoko Koga, Megumi Hara, Chisato Shimanoe, Yuichiro Nishida, Takuma Furukawa, Chiharu Iwasaka, Keitaro Tanaka, Jun Otonari, Hiroaki Ikezaki, Yoko Kubo, Yasufumi Kato, Takashi Tamura, Asahi Hishida, Keitaro Matsuo, Hidemi Ito, Yohko Nakamura, Miho Kusakabe, Daisaku Nishimoto, Keiichi Shibuya, Sadao Suzuki, Miki Watanabe, Etsuko Ozaki, Daisuke Matsui, Kiyonori Kuriki, Naoyuki Takashima, Aya Kadota, Kokichi Arisawa, Sakurako Katsuura-Kamano, Kenji Takeuchi, Kenji Wakai

**Affiliations:** 1 Department of Preventive Medicine, Faculty of Medicine, Saga University, Saga, Japan; 2 Department of Nursing, Faculty of Medicine, Fukuoka University, Fukuoka, Japan; 3 Department of Pharmacy, Saga University Hospital, Saga, Japan; 4 Advanced Comprehensive Functional Recovery Center, Saga University Hospital, Saga, Japan; 5 Department of Psychosomatic Medicine, Graduate School of Medical Sciences, Kyushu University, Fukuoka, Japan; 6 Department of Psychosomatic Medicine, International University of Health and Welfare Narita Hospital, Chiba, Japan; 7 Department of Comprehensive General Internal Medicine, Graduate School of Medical Sciences, Kyushu University, Fukuoka, Japan; 8 Department of General Internal Medicine, Kyushu University Hospital, Fukuoka, Japan; 9 Department of Preventive Medicine, Nagoya University Graduate School of Medicine, Nagoya, Japan; 10 Division of Cancer Epidemiology and Prevention, Aichi Cancer Center Research Institute, Nagoya, Japan; 11 Division of Cancer Epidemiology, Nagoya University Graduate School of Medicine, Nagoya, Japan; 12 Division of Cancer Information and Control, Aichi Cancer Center Research Institute, Nagoya, Japan; 13 Division of Descriptive Cancer Epidemiology, Nagoya University Graduate School of Medicine, Nagoya, Japan; 14 Cancer Prevention Center, Chiba Cancer Center Research Institute, Chiba, Japan; 15 Department of International Island and Community Medicine, Kagoshima University Graduate School of Medical and Dental Sciences, Kagoshima, Japan; 16 School of Health Sciences, Faculty of Medicine, Kagoshima University, Kagoshima, Japan; 17 Department of Intensive Care Medicine, Kagoshima University Hospital, Kagoshima, Japan; 18 Department of Public Health, Nagoya City University Graduate School of Medical Sciences, Nagoya, Japan; 19 Department of Epidemiology for Community Health and Medicine, Kyoto Prefectural University of Medicine, Kyoto, Japan; 20 Laboratory of Public Health, Division of Nutritional Sciences, School of Food and Nutritional Sciences, University of Shizuoka, Shizuoka, Japan; 21 Department of Public Health, Kindai University Faculty of Medicine, Osaka-Sayama, Japan; 22 Department of Public Health, Shiga University of Medical Science, Otsu, Japan; 23 Department of Preventive Medicine, Tokushima University Graduate School of Biomedical Sciences, Tokushima, Japan

**Keywords:** Lifestyle modification, Chronic kidney disease

## Abstract

Elucidating the risk factors for chronic kidney disease is important for preventing end-stage renal disease and reducing mortality. However, little is known about the roles of psychosocial stress and stress coping behaviors in deterioration of the renal function, as measured by the estimated glomerular filtration rate (eGFR). This cross-sectional study of middle-aged and older Japanese men (n = 31,703) and women (n = 38,939) investigated whether perceived stress and coping strategies (emotional expression, emotional support seeking, positive reappraisal, problem solving, and disengagement) were related to the eGFR, with mutual interactions. In multiple linear regression analyses adjusted for age, area, lifestyle factors, and psychosocial variables, we found a significant inverse association between perceived stress and the eGFR in men (*P*_trend_ = 0.02), but not women. This male-specific inverse association was slightly attenuated after adjustment for the history of hypertension and diabetes and was more evident in lower levels of emotional expression (*P*_interaction_ = 0.003). Unexpectedly, problem solving in men (*P*_trend_ < 0.001) and positive reappraisal in women (*P*_trend_ = 0.002) also showed an inverse association with the eGFR. Perceived stress may affect the eGFR, partly through the development of hypertension and diabetes. The unexpected findings regarding coping strategies require the clarification of the underlying mechanisms, including the hormonal and immunological aspects.

## Introduction

The number of patients with end-stage renal disease (ESRD) requiring dialysis is increasing worldwide, and has become a major economic burden for medical care systems^1^. Worldwide, the number of chronic dialysis patients exceeded 370 million in 2016. As of the end of 2017, there were more than 330,000 chronic dialysis patients in Japan^2^. According to country-specific data of the United States Renal Data System, after Taiwan, Japan had the second highest prevalence of chronic dialysis patients^3^. The annual medical care cost of dialysis in Japan is estimated to be JPY 1.6 trillion, which is approximately 4% of the total medical care cost. The increasing number of dialysis patients, together with the declining birthrate and aging population, can make it difficult to run social security services through the current taxation system in this country^4^.

Chronic kidney disease (CKD) represents a disease entity with a gradual loss of the renal function, which is usually measured by the estimated glomerular filtration rate (eGFR), accompanied by kidney damage (e.g., increased urinary albumin). All people with an eGFR < 60 ml/min/1.73 m^2^ for three months are regarded as having CKD^5^. Since ESRD is preceded by worsening CKD, the early diagnosis and management of CKD is critical for the prevention of ESRD. The number of CKD patients in Japan is approximately 13.3 million, which is 13% of the total adult population. This means that approximately one in eight Japanese adults has CKD^6^. CKD patients are also at high risk for developing cardiovascular disease (CVD), which leads to increased mortality^7^. The elucidation of risk factors for CKD is important for both preventing ESRD and reducing mortality.

The major causes of CKD include diabetes, hypertension, and glomerulonephritis^8^. Lifestyle risk factors for CKD include smoking, drinking alcohol, obesity, and low physical activity^9^. Little is known about the association between psychosocial factors and CKD or the eGFR. Depression has been associated with the development of mild to moderate CKD in African-Americans^10^ and with decreased eGFR values in Taiwanese patients with CKD^11^. As for psychosocial stress and stress coping strategies, the available data are particularly limited. One study of African-Americans, which had a small sample size, reported no significant relationship between perceived stress and the eGFR^12^. Another study showed that higher levels of goal-striving stress were significantly associated with a greater prevalence of CKD and lower eGFR values^13^. To our knowledge, no studies on stress coping strategies and CKD/eGFR have been reported. Some coping strategies (e.g., adaptive coping strategies such as problem solving^14,15^) resulting from increased psychosocial stress might mitigate the effect (if any) of stress on the renal function. Further studies are needed to investigate the association of psychosocial stress and coping strategies with CKD or the renal function.

The current study aimed to investigate whether perceived stress and coping strategies were associated with the renal function in middle-aged and older Japanese men and women and whether the level of each coping strategy modified the association between perceived stress and the renal function. Although the association between each psychosocial factor and the renal function could be bilateral (i.e., psychosocial factors influence the renal function, and vice versa), we were interested in the effects of psychosocial factors on the renal function. We used the continuous measurement of the eGFR as the main outcome instead of categorizing subjects according to the presence or absence of CKD (e.g., eGFR < 60 vs. ≥ 60 ml/min/1.73 m^2^) to avoid losing the information of that measure.

## Methods

### Study subjects

The study subjects consisted of the participants in the Japan Multi-Institutional Collaborative Cohort (J-MICC) Study, which is an ongoing prospective cohort study undertaken in collaboration with universities/research institutions throughout Japan^16,17^. The J-MICC Study examines how lifestyle habits and genetic factors (e.g., genetic variants) mutually affect the occurrence of lifestyle-related diseases, mainly cancer, after tracking approximately 100,000 subjects for 20 years. It was launched in specific areas of Japan (Chiba, Shizuoka-Sakuragaoka, Shizuoka, Okazaki, Aichi Cancer Center, Daiko, Iga, Takashima, Kyoto, Tokushima, Fukuoka, Saga, Kagoshima, and the Kyusyu and Okinawa Population Study area) in 2005^17^. By the end of 2014, 92,530 men and women of 35–69 years of age were recruited. Most participants were community residents and health check-up examinees. From 92,530 participants in the baseline survey, we excluded subjects with the following conditions: missing data on perceived stress (n = 1,810) or serum creatinine (n = 19,936), creatinine levels of < 0.2 or > 2.0 mg/dl (n = 108), or a history of renal disease (n = 34). Consequently, the 70,642 remaining subjects were included in the analysis. The dataset used in the present study was fixed on March 12, 2020.

The study protocol was approved by ethical committees of Nagoya University Graduate School of Medicine and all collaborating universities/institutions (Aichi Cancer Center, Chiba Cancer Center, Nagoya City University Graduate School of Medical Sciences, Shiga University of Medical Science, Kyoto Prefectural University of Medicine, Kyushu University Graduate School of Medical Sciences, Saga University Faculty of Medicine, Kagoshima University Graduate School of Medical and Dental Sciences, Tokushima University Graduate School of Biomedical Sciences, and University of Shizuoka). Written informed consent was obtained from all participants. The present study was conducted according to the principles expressed in the World Medical Association Declaration of Helsinki.

### Study design

This cross-sectional study used data from the baseline survey of the J-MICC Study. The baseline assessment included data collection using a self-administered questionnaire on demographic characteristics (age and gender), psychosocial factors (perceived stress and coping strategies), lifestyle factors (drinking and smoking habits, physical activity, sleeping hours, and dietary habits), and disease history, as well as physical measurements (height and weight) and blood collection.

#### Psychosocial factors

Perceived stress was assessed asking “How much stress did you feel during the last year?” The subjects were requested to select one of the following answers: (1) “I felt no stress at all,” (2) “I felt little stress,” (3) “I felt moderate stress,” and (4) “I felt much stress.” The level of perceived stress was classified as low for answers (1) and (2), medium for answer (3), and high for answer (4). We used these three levels of perceived stress as categorical or ordinal variables in the subsequent analyses. Although the above measurement of perceived stress is simple, it showed fair one-year reproducibility (weighted κ = 0.55)^18^ and was significantly associated with stress-related behaviors (e.g., coping strategies, smoking, physical activity, and sleeping hours^18^) and urinary cortisol levels^19^.

For coping strategies, we used five items selected from a dispositional version of the General Coping Questionnaire^20^ or the Brief Coping Orientation to Problems Experienced^21^. After the query, “How do you cope with various problems and unfavorable events you experience in daily life?” subjects were requested to answer the frequency (four response categories: “seldom,” “sometimes,” “often,” and “very often”) of each of the following coping strategies: (1) “I express my negative feelings and thoughts” (termed ‘emotional expression’); (2) “I consult with someone close and ask him/her for encouragement” (termed ‘emotional support seeking’ [ESS]); (3) “I try to interpret the problem in a favorable way” (termed ‘positive reappraisal’); (4) “I try hard to solve the problem” (termed ‘problem solving’); and (5) “I let the problem take its own course” (termed ‘disengagement’). The level of each coping strategy was classified as low for the frequency of “seldom”, medium for "sometimes", and high for “often” or “very often”. These three levels were used as categorical or ordinal variables for each coping strategy. The one-year reproducibility, as estimated by the weighted κ statistic, was reported to be 0.41 for emotional expression, 0.49 for ESS, 0.30 for positive reappraisal, 0.48 for problem solving, and 0.31 for disengagement^18^.

#### Covariates

For drinking habit, subjects were classified into never, former, and current drinkers and for current drinkers, total ethanol consumption (g/day) was estimated from the reported consumption frequency and the amounts of alcoholic beverages, as well as beverage-specific ethanol concentrations. Smoking status was categorized as never, former, or current smoker, with further classification by the number of cigarettes per day. Physical activity was estimated as the metabolic equivalent (MET)-hours per week, based on the frequency and duration of daily and leisure time activities^22^. Sleeping hours per day was asked in an open-ended manner. Energy intake was estimated using a validated short food frequency questionnaire^23^. Subjects were considered to have a history of hypertension, diabetes, or hyperlipidemia if they currently had these conditions or if they had been diagnosed with or treated for these conditions by physicians. Height and weight were measured on the day of the survey and the body mass index (BMI) was calculated as the weight in kilograms divided by the square of height in meters (kg/m^2^).

#### Estimation of the eGFR

Venous blood was collected for the determination of creatinine and other biochemical measurements on the day of the survey. Serum creatinine was measured at external laboratories using an enzymatic method^24^. The eGFR (mL/min/1.73 m^2^) was estimated using the following formula, taking into account serum creatinine (mg/dl), age (years), and gender: 194 × creatinine ^−1.094^ × age ^−0.287^ for men and this estimate was multiplied by 0.739 for women^25^.

### Statistical analysis

Statistical analyses were performed using SAS (Ver. 9.4 for Windows; SAS Institute, Cary, NC, USA). All analyses were conducted by gender with adjustment for age because age was strongly associated with both the exposures (e.g., perceived stress) and the outcome (i.e., eGFR), and thereby exerted a large confounding effect. We examined possible associations of perceived stress, coping strategies, and eGFR with covariates using the age-adjusted Spearman's rank correlation coefficient (ρ). In our main analyses, multiple regression models were run with the eGFR as a dependent variable and each of perceived stress and coping strategies as the main independent variables. The *P* value for trend was based on the statistical significance of each psychosocial variable as an ordinal variable. The following four models were constructed: (1) Model 1 was adjusted for age and study area, (2) Model 2 was additionally adjusted for lifestyle factors (drinking, smoking, physical activity, sleeping hours, energy intake, and BMI), (3) Model 3 was additionally adjusted for perceived stress and coping strategies, and (4) Model 4 was additionally adjusted for the history of hypertension, diabetes, and hyperlipidemia. We regarded the results in Model 3 as the main effects of psychosocial variables because the history of hypertension, diabetes, and hyperlipidemia included in Model 4 may represent the main mediators linking these variables to the eGFR.

When a significant association was found between a psychosocial variable and the eGFR in the above analyses, we estimated the adjusted mean (and 95% confidence interval [CI]) of the eGFR according to that variable in both genders, with the LSMEANS statement of the GLM procedure of SAS. We also examined whether an interaction existed between perceived stress and each coping strategy and the eGFR by including a corresponding interaction term in the above multiple regression models. When a significant interaction was detected, a stratified analysis was conducted to estimate the adjusted mean eGFR according to perceived stress and an identified coping strategy. All *P* values reported were two-tailed, and *P* values of < 0.05 were considered statistically significant.

## Results

Of the 70,642 subjects, 44.9% (n = 31,703) were men. The mean age of the men and women subjects was 56.0 years and 55.2 years, respectively. Table 1 shows the characteristics of the study subjects according to gender. Regarding psychosocial factors, women had higher levels of perceived stress, ESS, positive reappraisal, and disengagement, whereas men showed higher levels of problem solving. A history of hypertension or diabetes was more frequently reported by men than by women. The average eGFR (ml/min/1.73 m^2^) was 76.3 in men and 80.0 in women.

**Table 1:** Characteristics of the study subjects by gender.

	Men	Women
	n = 31,703	n = 38,939
Age (years)	56.0 [9.2]	55.2 [9.2]
Drinking, n (%)		
Never drinker	6,400 (20.2)	24,025 (61.7)
Former drinker	1,081 (3.4)	737 (1.9)
Current drinker		
0.1–22.9 g/day	14,649 (46.2)	11,824 (30.4)
23.0–45.9 g/day	5,181 (16.3)	1,068 (2.7)
46.0 + g/day	3,956 (12.5)	391 (1.0)
Smoking, n (%)		
Never smoker	9,251 (29.2)	33,643 (86.4)
Former smoker	12,984 (41.0)	2,624 (6.7)
Current smoker		
1–19 cigarettes/day	2,944 (9.3)	1,709 (4.4)
20 + cigarettes/day	5,613 (17.7)	850 (2.2)
40 + cigarettes/day	857 (2.7)	41 (0.1)
Physical activity (MET-hours/week)	15.7 [14.6]	14.8 [12.8]
Sleeping hours (per day)	6.8 [1.0]	6.5 [1.0]
Energy intake (kcal/day)	1930.3 [375.5]	1536.4 [261.4]
Body mass index (kg/m^2^)	23.8 [3.1]	22.4 [3.4]
Perceived stress, n (%)		
Low	10,560 (33.3)	7,870 (20.2)
Medium	14,615 (46.1)	19,385 (49.8)
High	6,528 (20.6)	11,684 (30.0)
Coping strategy, n (%)		
Emotional expression		
Low	6,609 (20.8)	7,455 (19.1)
Medium	18,986 (59.9)	23,862 (61.3)
High	6,066 (19.1)	7,573 (19.4)
Emotional support seeking		
Low	16,887 (53.3)	8,810 (22.6)
Medium	11,966 (37.7)	19,023 (48.9)
High	2,786 (8.8)	11,040 (28.4)
Positive reappraisal		
Low	3,724 (11.7)	3,118 (8.0)
Medium	11,605 (36.6)	14,103 (36.2)
High	16,309 (51.4)	21,640 (55.6)
Problem solving		
Low	2,840 (9.0)	3,501 (9.0)
Medium	10,401 (32.8)	14,913 (38.3)
High	18,395 (58.0)	20,434 (52.5)
Disengagement		
Low	6,670 (21.0)	5,540 (14.2)
Medium	15,705 (49.5)	19,343 (49.7)
High	9,228 (29.1)	13,939 (35.8)
Hypertension, n (%)	7,620 (24.0)	6,314 (16.2)
Diabetes, n (%)	2,862 (9.0)	1,353 (3.5)
Hyperlipidemia, n (%)	5,039 (15.9)	6,127 (15.7)
Creatinine (mg/dl)	0.8 [0.1]	0.6 [0.1]
eGFR (ml/min/1.73 m^2^)	76.3 [14.0]	80.0 [14.9]

Data represent the mean [standard deviation] or number (percentage).

Some data were missing for drinking (number of men/number of women, 436/894), smoking (54/72), physical activity (20/22), body mass index (3/5), emotional expression (42/49), emotional support seeking (64/66), positive reappraisal (65/78), problem solving (67/91), disengagement (100/117), hypertension (61/64), diabetes (53/52), and hyperlipidemia (101/108).

Table 2 shows the age-adjusted Spearman’s rank correlation coefficients of psychosocial factors and eGFR with covariates according to gender. Perceived stress showed significant inverse correlations with physical activity (in men only), sleeping hours, and BMI and weak positive correlations with drinking and a history of hypertension, diabetes, and hyperlipidemia. In comparison to perceived stress, all coping strategies generally demonstrated weaker correlations with covariates. In men, correlations between the eGFR and smoking (ρ = 0.14), physical activity (ρ = 0.11), and BMI (ρ = − 0.11) were relatively evident whereas correlations between the eGFR and a history of hypertension (ρ = − 0.07), diabetes (ρ = 0.05), and hyperlipidemia (ρ = − 0.07) were relatively weak. In comparison to men, the eGFR-covariate correlations of women were similar in direction but weaker in magnitude.

**Table 2:** Age-adjusted Spearman’s rank correlation coefficients of perceived stress, coping strategies, and eGFR with covariates by gender.

	Age^a^	Drinking	Smoking	Physical activity	Sleeping hours	Energy intake	Body mass index	Hyper-tension	Diabetes	Hyper-lipidemia
**Men (n = 31,703)**										
Perceived stress	**− 0.31**	**0.01**	**− 0.02**	**− 0.09**	**− 0.13**	− 0.01	**− 0.02**	**0.04**	**0.02**	**0.06**
Coping strategy										
Emotional expression	**− 0.06**	**0.03**	**− 0.02**	**− 0.02**	**− 0.02**	**0.04**	0.00	**0.02**	0.00	**0.03**
Emotional support seeking	**− 0.18**	**0.03**	**− 0.03**	**0.02**	− 0.01	**0.06**	0.00	**0.01**	**− 0.02**	0.01
Positive reappraisal	**− 0.04**	**0.05**	**− 0.02**	− 0.01	**− 0.01**	0.01	**0.03**	0.00	**− 0.01**	0.00
Problem solving	**− 0.09**	**0.05**	− 0.01	**− 0.03**	**− 0.04**	0.00	**0.03**	**0.01**	0.00	**0.02**
Disengagement	**− 0.09**	0.00	**− 0.03**	− 0.01	0.00	**0.02**	**− 0.04**	0.00	0.00	**0.01**
eGFR	**− 0.28**	**0.01**	**0.14**	**0.11**	**0.02**	**0.05**	**− 0.11**	**− 0.07**	**0.05**	**− 0.07**
**Women (n = 38,939)**										
Perceived stress	**− 0.20**	**0.03**	**0.03**	0.01	**− 0.12**	0.00	**− 0.05**	**0.02**	**0.02**	**0.05**
Coping strategy										
Emotional expression	**− 0.15**	**0.03**	**0.02**	**− 0.02**	**0.02**	**0.03**	**− 0.03**	− 0.01	**− 0.01**	**0.02**
Emotional support seeking	**− 0.23**	**0.02**	**− 0.02**	**0.02**	**0.01**	**0.05**	**− 0.02**	0.01	− 0.01	**0.02**
Positive reappraisal	− 0.01	**0.04**	0.00	**0.02**	− 0.01	**0.02**	0.00	**− 0.02**	− 0.01	0.00
Problem solving	**− 0.06**	**0.02**	0.00	**0.04**	**− 0.02**	**0.02**	0.00	− 0.01	− 0.01	**0.01**
Disengagement	**− 0.10**	**0.03**	− 0.01	**− 0.01**	**0.02**	**0.02**	**− 0.04**	**− 0.01**	− 0.01	0.01
eGFR	**− 0.30**	− 0.01	**0.02**	**0.04**	0.01	**0.02**	**− 0.02**	**− 0.03**	**0.05**	**− 0.02**

Categorical variables (perceived stress, coping strategies, drinking, and smoking) were converted to ordinal variables. Bold font represents *P* < 0.05.

^a^Without adjustment.

**Figure 1:**
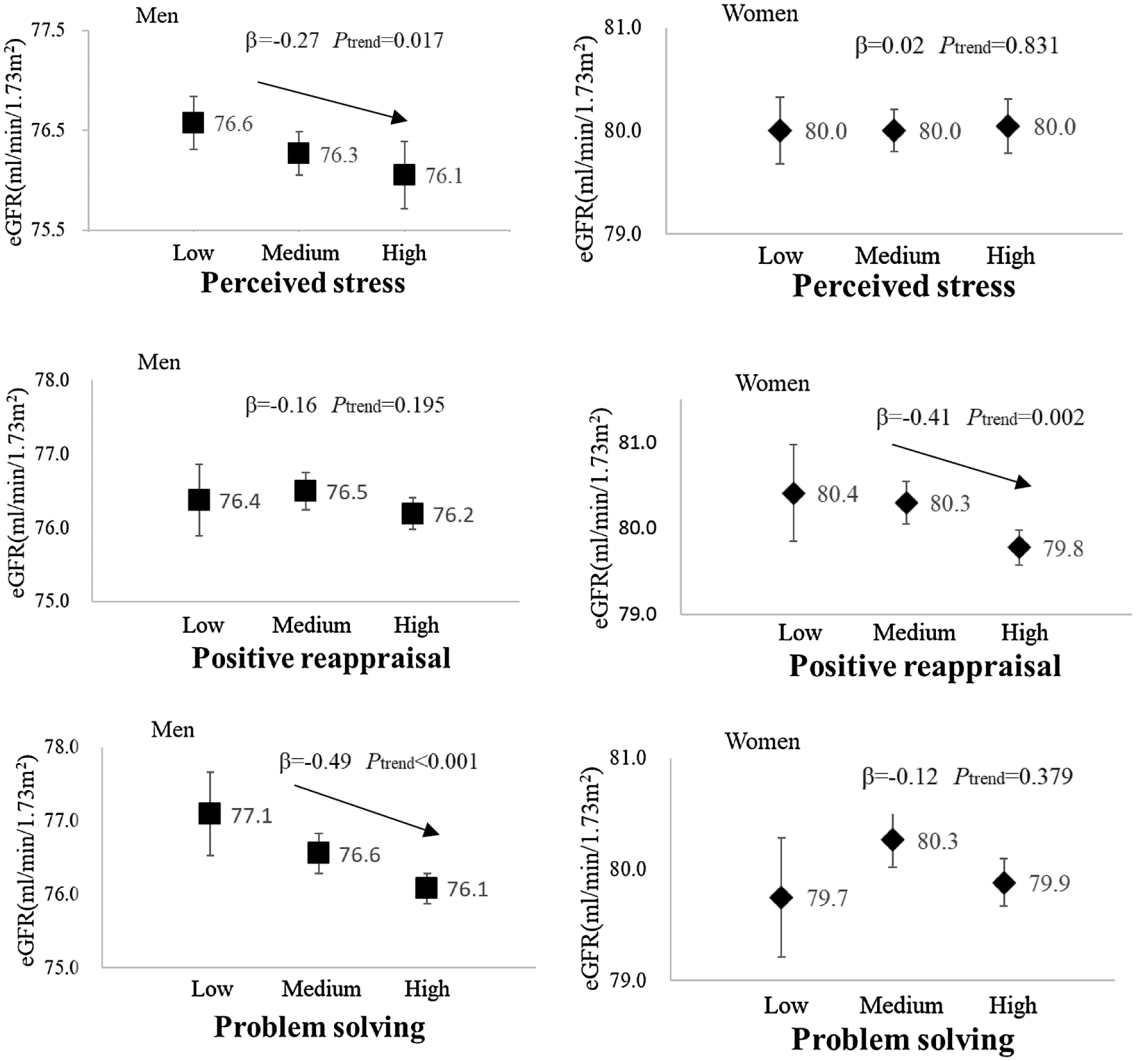
The adjusted mean eGFR values for each level of perceived stress, problem solving, and positive reappraisal according to gender. Symbols show averages and error bars represent the 95% confidence intervals. Adjustments were made for age, area, lifestyle factors (drinking, smoking, physical activity, sleeping hours, energy intake, and body mass index), and psychosocial factors (perceived stress and all coping strategies). β represents the regression coefficient of each psychosocial factor as an ordinal variable and P for trend denotes the statistical significance of β (see Model 3 in Table 3 for details).

Table 3 demonstrates the results from multiple regression analyses of the associations between eGFR and perceived stress and coping strategies, according to gender. After adjustment for age and area (Model 1), perceived stress in men (β = − 0.39, *P*_trend_ < 0.001), positive reappraisal in both men (β = − 0.45, *P*_trend_ < 0.001) and women (β = − 0.44, *P*_trend_ < 0.001), and problem solving in both men (β =  − 0.67, *P*_trend_ < 0.001) and women (β = − 0.24, *P*_trend_ = 0.026) were significantly inversely associated with the eGFR. These associations were slightly attenuated in men and remained unchanged in women after additional adjustment for lifestyle factors (Model 2). Further adjustment for psychosocial factors (Model 3, R^2^ = 0.13 for men and 0.12 for women) rendered the associations with positive reappraisal in men and problem solving in women non-significant and left perceived stress in men (β = − 0.27, *P*_trend_ = 0.017), problem solving in men (β = − 0.49, *P*_trend_ < 0.001), and positive reappraisal in women (β = − 0.41, *P*_trend_ = 0.002) as significant predictors of the eGFR (Fig. 1). After controlling for the history of hypertension, diabetes, and hyperlipidemia (Model 4, R^2^ = 0.14 for men and 0.13 for women), perceived stress in men (β = − 0.23, *P*_trend_ = 0.042), problem solving in men (β = − 0.45, *P*_trend_ < 0.001), and positive reappraisal in women (β = − 0.43, *P*_trend_ < 0.001) remained significant, although some attenuation in the strength of association was observed for perceived stress in men.

**Table 3:** Multiple regression analyses of the associations of perceived stress and coping strategies with eGFR by gender.

	Model 1^a^			Model 2^b^			Model 3^c^			Model 4^d^		
	β	SE	*P*_trend_^e^	β	SE	*P*_trend_^e^	β	SE	*P*_trend_^e^	β	SE	*P*_trend_^e^
Men (n = 31,703)	n = 31,703^f^			n = 31,202^f^			n = 31,015^f^			n = 30,865^f^		
Perceived stress	− 0.39	0.11	** < 0.001**	− 0.30	0.11	**0.006**	− 0.27	0.11	**0.017**	− 0.23	0.11	**0.042**
Coping strategy												
Emotional expression	− 0.13	0.12	0.277	− 0.15	0.12	0.207	− 0.06	0.12	0.632	− 0.05	0.12	0.657
Emotional support seeking	− 0.01	0.12	0.948	0.03	0.12	0.819	0.21	0.12	0.085	0.26	0.12	**0.029**
Positive reappraisal	− 0.45	0.11	** < 0.001**	− 0.39	0.11	** < 0.001**	− 0.16	0.13	0.195	− 0.19	0.13	0.129
Problem solving	− 0.67	0.12	** < 0.001**	− 0.59	0.11	** < 0.001**	− 0.49	0.14	** < 0.001**	− 0.45	0.14	** < 0.001**
Disengagement	− 0.01	0.11	0.903	− 0.01	0.11	0.930	− 0.08	0.11	0.455	− 0.09	0.11	0.425
Women (n = 38,939)	n = 38,939^f^			n = 37,981^f^			n = 37,723^f^			n = 37,587^f^		
Perceived stress	0.05	0.10	0.609	0.02	0.11	0.847	0.02	0.11	0.831	0.04	0.11	0.697
Coping strategy												
Emotional expression	− 0.15	0.12	0.184	− 0.15	0.12	0.196	− 0.17	0.12	0.159	− 0.16	0.12	0.194
Emotional support seeking	− 0.10	0.10	0.326	− 0.11	0.10	0.280	− 0.02	0.11	0.858	0.00	0.11	0.978
Positive reappraisal	− 0.44	0.11	** < 0.001**	− 0.44	0.11	** < 0.001**	− 0.41	0.13	**0.002**	− 0.43	0.13	** < 0.001**
Problem solving	− 0.24	0.11	**0.026**	− 0.28	0.11	**0.011**	− 0.12	0.13	0.379	− 0.09	0.13	0.477
Disengagement	0.02	0.11	0.837	0.01	0.11	0.905	− 0.02	0.11	0.837	− 0.02	0.11	0.861

Abbreviations: β, regression coefficient; SE, standard error. The multiple regression models included the eGFR as a dependent variable and each perceived stress and coping strategy as the main independent variables (ordinal variables). The unit of β and SE is ml/min/1.73 m^2^. Bold font represents *P* < 0.05.

^a^Adjusted for age and area.

^b^Additionally adjusted for drinking, smoking, physical activity, sleeping hours, energy intake, and body mass index.

^c^Additionally adjusted for perceived stress and all coping strategies. The R^2^ value was 0.13 for men and 0.12 for women.

^d^Additionally adjusted for the history of hypertension, diabetes, and hyperlipidemia. The R^2^ value was 0.14 for men and 0.13 for women.

^e^Represents the statistical significance of β.

^f^Denotes the number of subjects analyzed in the model including perceived stress as a main independent variable.

**Figure 2:**
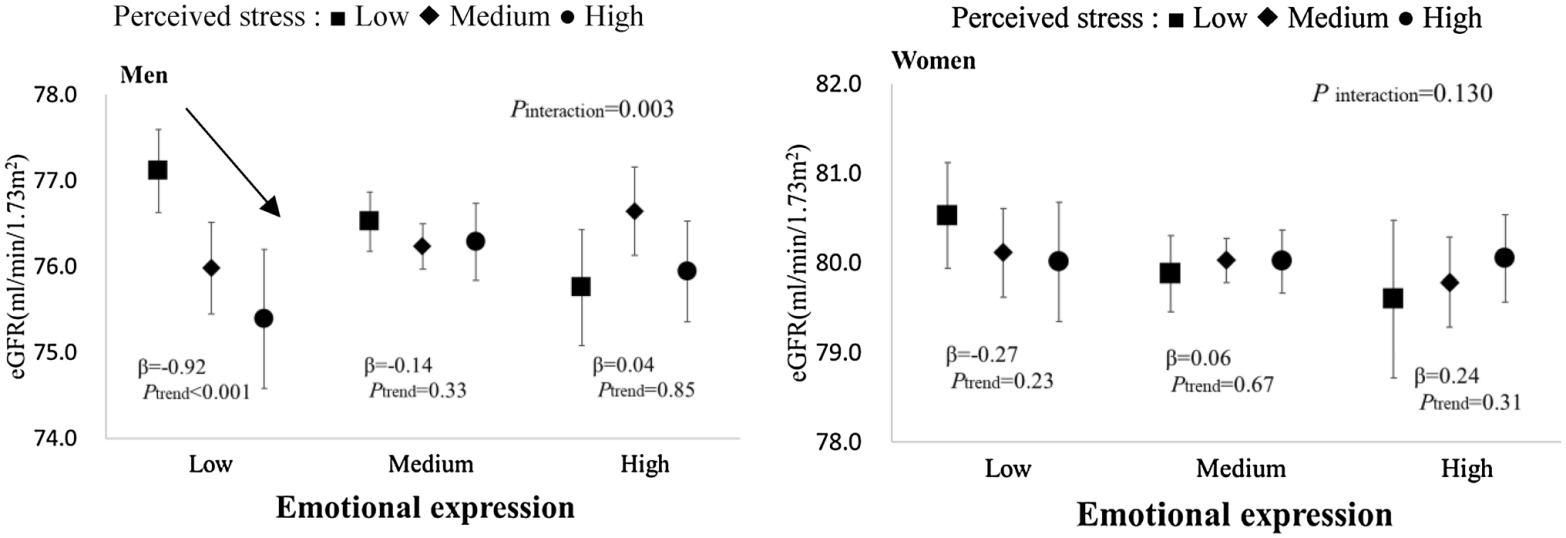
Adjusted mean eGFR values for perceived stress and emotional expression according to gender. Symbols show averages and error bars represent 95% confidence intervals. Adjustments were made for age, area, lifestyle factors (drinking, smoking, physical activity, sleeping hours, energy intake, and body mass index), and psychosocial factors (perceived stress and all coping strategies). β represents the regression coefficient of perceived stress as an ordinal variable and *P* for trend denotes the statistical significance of β.

We tested gender-specific interactions between each coping strategy and perceived stress on eGFR by including an additional interaction term in Model 3, as described above (Table 4). Accordingly, we found a significant interaction between emotional expression and perceived stress and the eGFR in men (*P*_interaction_ = 0.003). Figure 2 shows the adjusted mean of the eGFR according to the emotional expression and perceived stress in both genders. In men, an inverse association between perceived stress and eGFR was evident for the individuals with a low level of emotional expression (β = − 0.92, *P*_trend_ < 0.001), but not for those with a medium (β = − 0.14, *P*_trend_ = 0.33) or high (β = 0.04, *P*_trend_ = 0.85) level of emotional expression.

**Table 4:** *P* values for interactions between perceived stress and each coping strategy and eGFR by gender.

Coping strategy	Men	Women
Emotional expression	**0.003**	0.130
Emotional support seeking	0.675	0.951
Positive reappraisal	0.554	0.094
Problem solving	0.054	0.200
Disengagement	0.710	0.622

*P* values were derived from the interaction terms included in Model 3 as described in Table 3. Bold font represents *P* < 0.05.

## Discussion

In the present study, we found a statistically significant inverse association between perceived stress and the renal function (eGFR) in men, but not in women. Moreover, we unexpectedly discovered that problem solving in men and positive appraisal in women were also inversely associated with the eGFR. While perceived stress in men only had a weak association with the eGFR after adjustment for established risk factors (i.e., hypertension and diabetes), problem solving in men and positive reappraisal in women showed a robust inverse association with the eGFR after the same adjustment. To our knowledge, this is the first study to show that perceived stress and some coping strategies are associated with the deterioration of the renal function in an Asian population.

As for psychosocial stress, a previous study of 129 African-Americans showed that there was no significant relationship between perceived stress and the eGFR^12^. Another large study of 4967 African-Americans demonstrated that an increase in one standard deviation of the goal-striving stress score was associated with a 0.71-unit decrease in the eGFR^13^. This decrease is larger than our estimate of a 0.27–0.39-unit decrease in the eGFR per one category increase of perceived stress in men (Table 3, Models 1–3). African-Americans are known to have a high risk of developing CKD, with psychosocial factors such as low levels of income, social status, and education reported as possibly relevant factors^26^, while the Japanese also have a high risk of developing CKD, as stated in the Introduction. In these two high-risk populations, psychosocial stress might deteriorate renal function to some different degree.

Elevated stress can exert various health effects through the activation of the hypothalamic-pituitary adrenal (HPA) axis and sympathetic nervous system^27^. Among these effects, hypertension and diabetes represent dominant risk factors for CKD, which may possibly mediate the association between perceived stress and a decreased eGFR^8^. Actually, the inverse association between perceived stress and the eGFR in this study, although still significant, was slightly attenuated after controlling for hypertension and diabetes (Table 3, Model 4). Since the history of hypertension and diabetes was based on self-reporting and were therefore inaccurate, residual mediating effects may have existed. Furthermore, elevated oxidative stress (e.g., increased urinary excretion of 8-hydroxydeoxygunosine) associated with perceived stress might contribute to the inverse association between perceived stress and the eGFR because oxidative stress has been implicated in the pathogenesis of CKD^28,29^. Regarding the absence of the above stress-eGFR association in female participants, previous studies showed that women tend to suppress the stress-induced response of the HPA axis or sympathetic nervous system in comparison to men^30,31^, which may account for the gender difference.

In the present study, we unexpectedly found that problem solving in men and positive reappraisal in women were significantly inversely associated with the eGFR, even after full adjustment. Both strategies are adaptive coping strategies^14,15^, which have been generally linked to favorable health outcomes^32^. Although the reasons for the above results are essentially unknown, we explored possible explanations as follows. Regarding the inverse association between problem solving and the eGFR in men, we extensively searched correlates of problem solving, which may also affect the renal function, using data from a sub-cohort with relevant biological and physiological measurements^33^. As a result, we found that problem solving in men was significantly positively correlated with grip strength (age-adjusted ρ = 0.07 [*P* < 0.001] based on 3384 male subjects, data not shown), which has been reported to be strongly associated with serum testosterone levels^34^. Since testosterone has been implicated in the deterioration of the renal function^35^, one possible explanation may be that men employing higher levels of problem solving tend to have higher testosterone levels, and thereby present a lower eGFR. Unfortunately, we do not have testosterone-related measurements; thus, this possibility requires further confirmation.

Positive reappraisal possibly influences the immune function rather than the HPA axis or the sympathetic-adrenal medullary axis^36^. In a Korean study of medical students, those with more positive reappraisal showed lower interleukin-2 (IL-2) production in comparison to those with less reappraisal, particularly during chronic stress periods^37^. IL-2 has both immunostimulative and immunosuppressive functions^37^, but the administration of low-dose IL-2 or IL-2/anti-IL-2 antibody immunocomplexes with an increased half-life of IL-2 led to an increase in circulating regulatory CD4 + T lymphocytes (Tregs), which protected against CKD in animal experiments^38^. Accordingly, individuals with more positive reappraisal might present a decreased eGFR due to the presence of smaller numbers of Tregs as a result of lower IL-2 levels. This hypothesis also warrants further validation.

In the present study, we found a significant interaction between emotional expression and perceived stress and the eGFR in men (Table 4). The inverse association between perceived stress and the eGFR in men was more evident in lower levels of emotional expression (Fig. 1, β = -0.92, -0.14 and 0.04 in low, medium and high levels of emotional expression, respectively). This finding suggests that the possible detrimental effect of stress on the renal function in men may be mitigated by higher levels of emotional expression. This type of interaction has attracted much attention in studies on psychosocial stress, coping strategies, and health-related outcomes and should be further explored in future studies, hopefully with a longitudinal design.

The present study was associated with several limitations. First, the cross-sectional design of this study makes it difficult to infer the causality between perceived stress/coping strategies and the renal function. Individuals with an advanced stage of CKD (e.g., dialysis patients) may exhibit high stress levels as a result of their disease^39^. However, such individuals were excluded from the analysis and reverse causation does not seem to be a likely explanation for the observed associations. Second, the levels of perceived stress and coping strategies were assessed by a simple questionnaire using subjective questions. Since our measurement of perceived stress has fair reproducibility and reasonable associations with stress-related behaviors and biomarkers, we believe that the observed findings reflect some aspects of the stress-eGFR relationship. However, direct comparisons between our results and those in other studies using different measurements may be difficult. For coping strategies, the one-year reproducibility as measured by the weighted κ ranged from 0.30 to 0.49^18^. This measurement error might have resulted in weaker associations but is unlikely to have altered the interpretation of the results. Third, the clinical significance of our findings is uncertain because even the strongest association with problem solving in men showed an approximately 0.5-unit decrease in eGFR per one category increase. However, the deteriorated renal function is usually irreversible and accumulating effects of weak risk factors may become substantial over the long-term during life. Fourth, the present study only included Japanese subjects, which makes it difficult to extrapolate the results and interpretations to non-Japanese subjects who have different psychosocial backgrounds.

## Conclusions

We found a statistically significant inverse association between perceived stress and the eGFR in men, but not in women. This male-specific association may be partly mediated by hypertension and diabetes resulting from elevated stress. Moreover, we unexpectedly observed that problem solving in men and positive reappraisal in women were inversely associated with the eGFR. The precise mechanisms of these unexpected findings, including hormonal and immunological aspects, remain to be elucidated.

## Data Availability

The datasets generated during and/or analyzed during the current study are not publicly available due to ethical restriction but are available from the corresponding author on reasonable request.
